# Factors associated with home births in Peru 2015–2017: A cross-sectional population-based study

**DOI:** 10.1016/j.heliyon.2021.e06344

**Published:** 2021-03-08

**Authors:** Akram Hernández-Vásquez, Horacio Chacón-Torrico, Rodrigo Vargas-Fernández, Guido Bendezu-Quispe

**Affiliations:** a Universidad San Ignacio de Loyola, Vicerrectorado de Investigación, Centro de Excelencia en Investigaciones Económicas y Sociales en Salud, Lima, Peru; b Universidad Científica del Sur, Lima, Peru; c Universidad Privada Norbert Wiener, Lima, Peru

**Keywords:** Delivery, Home childbirth, Obstetric, Parturition, Health surveys, Risk factors, Peru

## Abstract

**Background:**

Higher rates of maternal complications and deaths have been described in home births. However, few local studies have evaluated factors associated with home births in Peru. The study aims to determine the prevalence and factors associated with home birth in the Peruvian population.

**Methods:**

A population-based analytical cross-sectional study was conducted using pooled data from the 2015–2017 Peruvian Demographic and Health Surveys. A logistic regression model was performed to calculate crude and adjusted odds ratios (aOR) for the association between sociodemographic and mother-related factors and home births.

**Results:**

Seven out of every 100 births were home births. Living in a rural area (aOR = 3.10; 95% CI: 2.52–3.81), having a primary or secondary educational level, belonging to a medium or low wealth tertile, being from the rest of the Coast, Andean or Amazon regions, the second or greater number of birth order and considering the distance to the health center as problematic (aOR: 1.32; 95% CI: 1.17–1.48) were found to be associated with a higher probability of home births. Contrarily, being in the age groups of 25–34 and 35–39 years old, having a multiple pregnancy and giving birth to a medium (aOR: 0.88; 95% CI: 0.78–1.00) or large-sized newborn (aOR = 0.81; 95% CI: 0.72–0.93) were associated with a lower probability of presenting home births.

**Conclusions:**

Sociodemographic factors are associated with home births in Peru. Further study of these factors is required to develop strategies specific to the needs of the population of childbearing age.

## 1 Introduction

Globally, maternal mortality declined by 30% between 1990 and 2015, with 80% of maternal deaths during gestation, delivery, and postpartum taking place in countries belonging to the two bottom quintiles of the sociodemographic index [1]. In 2016, more than 2.5 million children died in the first month of life, most during the first week after birth [2]. For this reason, as part of the Sustainable Development Goal number three, the United Nations seeks to reduce maternal and infant mortality and improve maternal health worldwide [3].

Most maternal deaths occur during the intrapartum and the immediate puerperium [4]. Access to and use of maternal health services during childbirth have been reported to reduce maternal-perinatal deaths and complications due to skilled care, postnatal care, and monitoring during the immediate postpartum period [5, 6]. Throughout the world, there have been significant advances in the last two decades regarding the percentage of births attended by skilled health personnel, rising from 62% (2000–2005) to 81% (2013–2018) [7]. Despite this, in 2017, approximately 295,000 women in the world died from causes related to pregnancy and childbirth [8]. Although childbirth care in health facilities or accompanied by skilled health personnel is encouraged, in various regions of the world, especially in low-income countries, home births represent more than 50% of the total number of births [9, 10]. Higher rates of maternal and perinatal complications and deaths have been described in home births, as well as difficulties in managing complications due to the need for transfer to a health facility [11, 12, 13].

Various determinants of home births have been identified, including maternal age, parity, educational level, marital status, socioeconomic status, community health infrastructure, region and area of residence, and distance to the health center [14, 15]. Likewise, regional and socioeconomic differences related to home births have been studied, and the percentage of home births has been described to vary among the poorest quintile from 37.3% in Europe, North Africa, and the Middle East up to 88.5% in South Asia. Latin America is the most unequal region regarding the proportion of home births, with only 3.1% of home births for women in the richest quintile and 47.9% for women in the poorest quintile [16].

Peru has experienced a reduction in maternal mortality, decreasing from 265 to 68 maternal deaths per 100,000 live births in the period from 1990 to 2015 (i.e., the Millennium Goals timeline) [17], with an increase in institutionalized and skilled health personnel attended childbirth. However, deaths and complications related to pregnancy and childbirth continue to be a public health problem, characterized by unequal geographic and socioeconomic distribution [18]. Regarding home births in Peru, community studies with a low number of participants have described some associated factors, including the continuum and quality of prenatal care, distance to the health facility, and home birth planning [19, 20, 21]. Given the geographic variability and access to health services in Peru, it is necessary to study the factors associated with home births to develop effective interventions and design programs aimed at improving outcomes in maternal health. Therefore, the objective of this study was to determine the prevalence and factors associated with home births in the Peruvian population.

## 2 Methods

### 2.1 Study design and data sources

An analytical cross-sectional population-based study was carried out and included the pooled data from 2015, 2016, and 2017 Peruvian Demographic and Health Surveys (ENDES). ENDES is an annual survey that is representative of the Peruvian population, according to the area of residence (urban and rural) and natural region, and has been carried out by the National Institute of Statistics and Informatics (INEI) since 2004. In this regard, Peru is divided into three natural regions with different characteristics. The Coast region stretches along the Pacific Ocean and includes Lima, the country's capital. The Andean region is located in the Andean zone, where most of the cities within this region present difficult geographical access and low resources. Finally, the Amazon region corresponds to the Amazon territory, being the largest and least densely populated region in Peru.

One of the objectives of the ENDES survey is to obtain information on the health status of women of childbearing age (15–49 years). Every year, the ENDES survey collects data on all births in the last five years prior to the survey. For the purposes of this study, data from 56,594 women aged 15–49 years with data from the last birth in the last five years prior to the survey were considered. The specific sampling, processing, and data collection methods are detailed in the ENDES technical report [22].

### 2.2 Variables

The dependent variable was home birth, which was generated from variable M15 (place of birth) of the ENDES database, and was self-reported by the mother and recoded with “1” if the birth was at home and “0” when the birth was in another place (hospital/clinic/health facility/among others). Based on the literature on this topic [9, 10, 16, 23], the following characteristics of the mother were considered as independent variables: age group (in years), educational level (up to primary/secondary/higher), health insurance (yes/no), wealth tertile (low/medium/high) in which a low tertile corresponds to the poorest and a high tertile corresponds to the richest population, type of pregnancy, prenatal control (0–7/8 or more) and native language (non-native/native), characteristics of the newborn including gender (male/female), birth order (1/2/3/4 or more), size of the newborn (small/medium/large) and characteristics of the environment including area of residence (urban/rural) and geographic domain (Metropolitan Lima/rest of the Coast/Andean/Amazon).

### 2.3 Statistical analysis

The pooling of the survey databases corresponding to the years 2015, 2016, and 2017 was made considering that they have the same sampling frame. Absolute frequencies and weighted proportions with 95% confidence intervals (95% CI) were reported for the dependent and independent variables. The chi-square test was used to assess associations among the study variables. Then, the association between the independent variables and home births was evaluated using a logistic regression model to calculate the crude and adjusted odds ratios (OR) with their respective 95% CI. Variables with a p-value < 0.20 in the crude analysis were included in the multiple logistic regression model, and the Hosmer-Lemeshow test was used to evaluate the goodness of fit of the adjusted model. The variance inflation factors (VIF) were generated to diagnose multicollinearity among the independent variables. The highest-observed VIF was 2.22, and the lowest was 1.02, indicating a lack of serious multicollinearity. All hypotheses testing to determine differences and associations were judged significant at p < 0.05. Data cleaning, management, and analysis were carried out using Stata 14.2 (*Stata Corp, College Station, TX, US*). For all the analyses, the weighting factors and specifications of the complex sample design of ENDES were considered, using the svy command.

### 2.4 Ethical considerations

The performance of this study did not require the approval of an ethics committee as it was an analysis of secondary data available in the public domain that did not allow the identification of the respondents. The ENDES database is freely accessible and can be obtained at http://iinei.inei.gob.pe/microdatos/.

## 3 Results

A total of 56,594 women aged 15–49 years were included with complete data regarding their last birth in the last five years. Among the characteristics of the women, 74.4% lived in an urban area (Table 1). According to the natural region, 29.5% lived in Metropolitan Lima and 26.4% in the rest of the Coast region. Regarding age groups, most mothers were between 25 and 34 years old (46.4%). With respect to educational level, 21.9% of the women had received primary education, while the majority had up to a secondary educational level (46.7%). In relation to the wealth index, the distribution according to this characteristic was similar among all its categories. Only 4.6% of women spoke a native language, and 83.1% of women had health insurance. Regarding accessibility to health facilities, 37.2% considered that they had an access problem. Concerning the characteristics of the pregnancy, the highest proportion of women answered having had their first (32.8%) or second birth (31.1%). Relating to the newborn, most had a medium size at birth (50.2%), 51.2% were male, and 99% of births were singletons. Regarding care during pregnancy, 70.2% of women had eight or more antenatal controls (ANC).

**Table 1:** Background characteristics of respondents, Peru 2015–2017.

Characteristic	Unweighted number	Weighted percent (95% CI)†
**Total**	56 594	100

**Place of residence**		
Urban	40 093	74.4 (73.2–75.7)
Rural	16 501	25.6 (24.3–26.8)

**Natural region**		
Metropolitan Lima	6499	29.5 (27.2–31.9)
Rest of Coast	17 522	26.4 (24.6–28.2)
Andean	18 194	27.0 (25.3–28.8)
Amazon	14 379	17.1 (15.7–18.6)

**Wealth Index**		
High	15 398	34.3 (32.9–35.8)
Middle	20 273	34.0 (32.9–35.1)
Low	20 923	31.7 (30.4–33.0)

**Level of education**		
Higher	16 851	31.4 (30.4–32.5)
Secondary	26 570	46.7 (45.8–47.6)
Only elementary school	13 173	21.9 (21.0–22.8)

**Age groups, years, age of the mother at childbirth**		
15–17	936	1.5 (1.4–1.7)
18–24	13 612	23.1 (22.5–23.6)
25–34	26 227	46.4 (45.7–47.0)
35–49	15 819	29.1 (28.5–29.7)

**Order of birth**		
1	18 069	32.8 (32.2–33.4)
2	17 227	31.1 (30.5–31.7)
3	10 448	18.4 (18.0–18.9)
4 or more	10 850	17.7 (17.2–18.2)

**Newborn size**		
Small	11 986	21.2 (20.7–21.7)
Medium	29 054	50.2 (49.5–50.9)
Large	15 554	28.6 (28.0–29.2)

**Newborn gender**		
Female	27 662	48.8 (48.2–49.4)
Male	28 932	51.2 (50.6–51.8)

**Type of pregnancy**		
Single	56 056	99.0 (98.9–99.1)
Multiple	538	1.0 (0.9–1.1)

**Antenatal controls**		
0 to 7	16 976	29.2 (28.5–29.9)
8 or more	39 618	70.2 (70.1–71.5)

**Language learned in childhood**		
No native	53 100	95.4 (94.9–95.9)
Native	3494	4.6 (4.1–5.1)

**Health insurance**		
Yes	47 742	83.1 (82.6–83.7)
No	8852	16.9 (16.3–17.4)

**Distance**		
No problem	35 078	62.8 (61.9–63.7)
Problem	21 516	37.2 (36.3–38.1)

† Weighted percentages are according to sampling specifications and weights of ENDES by year.

Home births represented 6.8% of all childbirths. In rural areas, 21.5% of childbirths were domiciliary, while in urban areas, only 1.8% were home births (Table 2). The highest proportions of home births occurred in the Andean (10.3%) and Amazon (18.8%) regions. Regarding the characteristics of the mother, according to the wealth index, the highest proportion of home births occurred among women in the lowest tertile (19%), with the lowest proportion being in the middle (2.0%) and highest (0.4%) tertiles. The highest proportion of home births by age occurred in the 15–17 years of age group (10.9%). Women who had a primary education had the highest proportion of home births (21.0%). Likewise, women who spoke a native mother tongue had a higher proportion of home births (23.5%). Additionally, women who considered the distance to the health facility as being problematic also presented a higher prevalence of home births (11.5%). The percentage of home births was similar in women with (6.9%) or without health insurance (6.4%). In relation to the pregnancy characteristics, women with their fourth or higher birth presented the highest proportion (17.1%) of home births. Regarding newborn size, 9.5%, 6.4%, and 5.6% of the newborns were small, medium, and large, respectively. According to the sex of the newborn, 6.7% of female children and 7.0% of male children were born at home. Additionally, 6.9% of single pregnancies and 3.7% of multiple pregnancies corresponded to home births. Regarding ANCs, the proportion of home births in pregnancies with seven or fewer ANCs was 12.1%, being higher than that of women with eight or more controls (4.7%).

**Table 2:** Proportion of home births by characteristics of respondents, Peru 2015–2017.

Characteristics	Home birth		
	No	Yes	P-value†
	Weighted percent (95% CI)	Weighted percent (95% CI)	
**Place of residence**			
Urban	98.2 (97.9–98.1)	1.8 (1.6–2.1)	<0.001
Rural	78.5 (76.0–80.8)	21.5 (19.2–24.0)	

**Natural region**			
Metropolitan Lima	99.5 (99.2–99.7)	0.5 (0.3–0.8)	<0.001
Rest of Coast	97.4 (96.4–98.0)	2.6 (2.0–3.6)	
Andean	89.7 (88.0–91.2)	10.3 (8.8–12.0)	
Amazon	81.2 (78.3–83.8)	18.8 (16.2–21.7)	

**Wealth Index**			
High	99.6 (99.4–99.7)	0.4 (0.3–0.6)	<0.001
Middle	98.0 (97.6–98.3)	2.0 (1.7–2.4)	
Low	81.0 (79.0–82.9)	19.0 (17.1–21.0)	

**Level of education**			
Higher	99.2 (99.0–99.4)	0.8 (0.6–1.0)	<0.001
Secondary	95.7 (95.2–96.2)	4.3 (3.8–4.8)	
Only elementary school	79.0 (76.8–81.0)	21.0 (19.0–23.2)	

**Age groups, years, age of the mother at childbirth**			
15–17	89.1 (86.3–91.4)	10.9 (8.6–13.7)	<0.001
18–24	92.4 (91.3–93.3)	7.6 (6.7–8.7)	
25–34	93.5 (92.7–94.2)	6.5 (5.8–7.3)	
35–49	93.5 (92.6–94.2)	6.5 (5.8–7.4)	

**Order of birth**			
1	99.6 (96.1–97.1)	3.4 (2.9–3.9)	<0.001
2	95.2 (94.6–95.7)	4.8 (4.3–5.4)	
3	93.3 (92.5–94.1)	6.7 (5.9–7.5)	
4 or more	82.9 (80.8–84.8)	17.1 (15.2–19.2)	

**Newborn size**			
Small	90.5 (89.1–91.7)	9.5 (8.3–10.9)	<0.001
Medium	93.6 (92.0–94.2)	6.4 (5.8–7.1)	
Large	94.4 (93.5–95.2)	5.6 (4.8–6.5)	

**Newborn gender**			
Female	93.3 (92.5–94.0)	6.7 (6.0–7.5)	0.400
Male	93.0 (92.2–93.8)	7.0 (6.2–7.8)	

**Type of pregnancy**			
Single	93.1 (92.4–93.8)	6.9 (6.2–7.6)	0.046
Multiple	96.3 (93.1–98.0)	3.7 (2.0–6.9)	

**Antenatal controls**			
0 to 7	87.9 (86.5–89.3)	12.1 (10.7–13.7)	<0.001
8 or more	95.3 (94.7–95.8)	4.7 (4.2–5.3)	

**Language learned in childhood**			
No native	94.0 (93.2–94.6)	6.0 (5.4–6.8)	<0.001
Native	76.5 (71.2–81.1)	23.5 (18.9–28.8)	

**Health insurance**			
Yes	93.1 (92.3–93.8)	6.9 (6.2–7.7)	0.387
No	93.6 (92.3–94.6)	6.4 (5.4–7.7)	

**Distance**			
No problem	95.9 (95.4–96.4)	4.1 (3.6–4.6)	<0.001
Problem	85.5 (87.1–89.8)	11.5 (10.2–12.9)	

Weighted percentages of the row are according to sampling specifications and weights of ENDES by year.

† The p value was calculated using the Chi-square test.

With respect to the factors associated with home births (Table 3), it was found that living in rural areas (aOR = 3.10; 95% CI: 2.52–3.81) increased the probability of home births. Compared to residing in the metropolitan Lima area, home birth was more likely if the women were from the rest of the natural Coast (aOR = 1.68; 95% CI: 0.98–2.87), Andean (aOR = 2.60; 95% CI: 1.52–4.42) or Amazon regions (aOR = 5.32; 95% CI: 3.18–8.91). Women in the age groups 25 to 34 (aOR = 0.63; 95% CI: 0.46–0.86) and 35–39 years old (aOR = 0.39; 95% CI: 0.28–0.54) were less likely to have had home births, compared to those between 15 and 17 years of age. Additionally, women in the medium (aOR = 1.73; 95% CI: 1.23–2.43) or low wealth index (aOR = 3.88; 95% CI: 2.72–5.55) were more likely to have home births compared to those from a high wealth index. Regarding the level of education, women with a primary (aOR = 3.15; 95% CI: 2.42–4.08) or secondary education (aOR = 1.52, 95% CI: 1.19–1.94) had a higher probability of home births compared to women with a higher educational level. The characteristics of the mother, such as having health insurance or if she learned a native language during childhood, were not found to be associated with home births.

**Table 3:** Factors associated with the home birth, Peru 2015–2017.

Characteristics	Home birth			
	OR	P-value	aOR∗	P-value
**Place of residence**				
Urban	Ref.		Ref.	
Rural	14.78 (12.13–18.01)	<0.001	3.10 (2.52–3.81)	<0.001

**Natural region**				
Metropolitan Lima	Ref.		Ref.	
Rest of Coast	5.42 (3.14–9.37)	<0.001	1.68 (0.98–2.87)	0.057
Andean	22.96 (14.15–37.26)	<0.001	2.60 (1.52–4.42)	<0.001
Amazon	46.32 (28.43–75.44)	<0.001	5.32 (3.18–8.91)	<0.001

**Wealth Index**				
High	Ref.		Ref.	
Middle	4.74 (3.46–6.49)	<0.001	1.73 (1.23–2.43)	0.002
Low	53.98 (39.10–74.53)	<0.001	3.88 (2.72–5.55)	<0.001

**Level of education**				
Higher	Ref.		Ref.	
Secondary	5.54 (4.32–7.09)	<0.001	1.52 (1.19–1.94)	0.001
Only elementary school	32.92 (25.56–42.41)	<0.001	3.15 (2.42–4.08)	<0.001

**Age groups, years, age of the mother at childbirth**				
15–17	Ref.		Ref.	
18–24	0.67 (0.53–0.86)	0.001	0.91 (0.69–1.20)	0.501
25–34	0.57 (0.45–0.72)	<0.001	0.63 (0.46–0.86)	0.004
35–49	0.57 (0.44–0.73)	<0.001	0.39 (0.28–0.54)	<0.001

**Order of birth**				
1	Ref.		Ref.	
2	1.45 (1.27–1.64)	<0.001	1.75 (1.49–2.04)	<0.001
3	2.06 (1.79–2.36)	<0.001	2.25 (1.86–2.72)	<0.001
4 or more	5.95 (5.22–6.78)	<0.001	3.90 (3.15–4.82)	<0.001

**Newborn size**				
Small	Ref.		Ref.	
Medium	0.65 (0.58–0.74)	<0.001	0.88 (0.78–1.00)	0.046
Large	0.56 (0.50–0.64)	<0.001	0.81 (0.72–0.93)	0.002

**Newborn gender**				
Female	Ref.		Ref.	
Male	1.04 (0.95–1.12)	0.400	No include	

**Type of pregnancy**				
Single	Ref.		Ref.	
Multiple	0.53 (0.28–1.00)	0.050	0.43 (0.21–0.89)	0.024

**Antenatal controls**				
0 to 7	Ref.		Ref.	
8 or more	0.36 (0.32–0.41)	<0.001	0.50 (0.45–0.57)	<0.001

**Language learned in childhood**				
No native	Ref.		Ref.	
Native	4.78 (3.61–6.32)	<0.001	1.09 (0.83–1.44)	0.533

**Health insurance**				
Yes	Ref.		Ref.	
No	0.92 (0.76–1.11)	0.387	No include	

**Distance**				
No problem	Ref.		Ref.	
Problem	3.03 (2.67–3.44)	<0.001	1.32 (1.17–1.48)	<0.001

OR: odds ratio; aOR: adjusted odds ratio for all the variables present in the column.

∗All variables with a value of p < 0.20 in the crude analysis were included and the area under the curve (AUC) was 0.896.

The weighting factor and sample specifications of ENDES by year were included for all analyses.

In relation to pregnancy characteristics, compared to the first childbirth, the second or greater number of birth order was associated with home births (p < 0.001 for all the categories included) (Table 3). Additionally, having received eight or more antenatal controls decreased the probability of home births (aOR = 0.50; 95% CI: 0.45–0.57). Having a multiple pregnancy decreased the probability of having a home birth (aOR = 0.43; 95% CI: 0.21–0.89). A medium (aOR: 0.88; 95% CI: 0.78–1.00) or large size (aOR = 0.81; 95% CI: 0.72–0.93) newborn was associated with a lower probability of presenting a home birth. Considering the distance to the health center as problematic was associated with a higher prevalence of home births (aOR = 1.32; 95% CI: 1.17–1.48). The sex of the newborn was not found to be associated with home births.

## 4 Discussion

This study sought to determine the prevalence of home births in Peruvian women and its associated factors. It was found that 7 out of every 100 births were domiciliary. Living in a rural area, having a lower educational level (primary or secondary), belonging to the medium or low wealth tertile, being from the rest of the Coast, Andean, or Amazon regions, having a second or greater number of birth order and considering the distance to the establishment as a problem were associated with a greater probability of home births. On the other hand, being a woman in the age groups of 25–34 and 35–39 years old, having a multiple pregnancy and giving birth to a medium- or large-sized newborn was associated with a lower probability of giving birth at home. Thus, interventions to reduce home births and increase childbirth care by skilled health personnel should consider the characteristics of pregnant women when designing strategies to improve provision and accessibility to health services.

Regarding the place of residence, living in a rural area was associated with a higher proportion of home births. The proportion of home births in rural areas compared to those of urban areas in low-income countries has been reported as being high, being mainly attributed to less geographic access and transportation difficulties for reaching health facilities, the infrastructure of which is usually not adequate for care [24, 25]. This is especially true when considering the geographical isolation of the Peruvian Amazon and that the wide dispersion of health facilities in this region is a barrier to effective uptake of health systems [26]. Likewise, the probability of home births was higher among women living in the Andean region, the Amazon, or the rest of the Coast, compared to Metropolitan Lima (capital of Peru). The Amazon and Andean regions also have the highest proportion of indigenous populations, and the lack of cultural adaptation during childbirth and the perinatal period, including the language barrier, favor women prioritizing traditional home birth rather than institutionalized childbirth [27], a factor that could contribute to the higher proportion of home births in these areas. In addition, in these regions, the use of traditional birth attendants is very common likely due to easier accessibility to midwives care and greater familiarity with traditional practices, thereby increasing the number of home births as described in Peru [28] and other countries in the region [29, 30].

The results of this study indicate that less than 10% of Peruvian women had a home birth during their last childbirth in the last five years. Although global data on home births are not available, the estimates reported in Peru are lower than those described in African and Asian countries (greater than 50%) [6, 9, 10]. In Peru, public health insurance covers delivery care for all women affiliated in the national territory, seeking to eliminate the direct costs of delivery care, promoting delivery care in hospitals [31]. Likewise, in recent decades, social programs have been implemented to reduce inequalities in access to health care. One example is the JUNTOS program, a cash-transfer program that benefits low-income families in rural and urban areas conditional on preventive maternal and child check-ups attendance. This program has increased the demand for preventive health services, including family planning and prenatal care among women of childbearing age [32, 33]. By favoring contact between citizens and the health system, these social programs could promote birth care in health facilities. However, there is a proportion of women that continues with their birth attention at home. Since compared to institutional births, home births present worse results in maternal-perinatal health, it is necessary to study what factors influence maternal decisions regarding home birth and the quality of care in order to promote attitudes and practices towards institutional birth, and thus, improve maternal-perinatal outcomes in the Peruvian population that still practices home birth.

Birth in older women was less likely to take place at home. Studies in Ethiopia and Uganda found that increased knowledge of complications or warning signs during pregnancy increases the degree of preparation for institutionalized childbirth in women [34, 35]. Similarly, a greater number of ANC decreased the probability of home births. These findings could be explained by older women or health personnel placing greater emphasis on the benefits of childbirth in health facilities by skilled health personnel due to the increased risk of complications due to the mother's older age. However, a study in Nepal reported that older pregnant women had a higher probability of home births given the association between higher maternal age with multiparity and precipitous childbirth [36]. Nonetheless, in our study, women in the age group of young adolescents presented the highest probability of having a home birth. Hence, knowing the risks of home births, it is necessary to study in-depth the factors that determine the use of maternal health services for the care of births in this population subgroup.

The greater the birth order number, the more likely the birth took place at home. Previous studies in low and middle-income countries found an inverse relationship between the number of childbirths and institutional childbirth [24, 37]. Our study presents a panorama in which the primiparous are those with the highest home births, which could be associated with having less access to pregnancy-related information given their condition as a first-time mother, requiring and perceiving greater safety in a family environment such as the home or fear of the use of health services. On the other hand, having a multiple pregnancy was found to be associated with a lower probability of home births. In this regard, in various regions of the world, the indication of elective cesarean sections for multiple pregnancies is frequent due to possible complications that may occur during labor [38] despite the benefits of this procedure being inconclusive [39]. The indication of cesarean section in a multiple pregnancy decreases the probability of home births, which may, to some extent, explain this finding. In addition, having a medium or large newborn was associated with a lower probability of home births. This finding has also been reported in Bangladesh [40] and sub-Saharan countries [41]. In line with multiple pregnancies, a larger newborn would have a greater probability of indicating elective cesarean section in health facilities, which would reduce the probability of home births.

Considering the distance to the health facility as a problem was associated with a higher proportion of home births. In Peru, previous studies carried out in the Andean and Amazon communities described that living at a distance greater than 90 min from the health facility was associated with a greater probability of home births [20]. Studies in countries in different regions of the world have reported that the accessibility to health facilities is associated with the use of health services for childbirth care [42, 43]. Thus, poor geographic accessibility to health facilities conditions a home birth without skilled health personnel [42]. Since accessibility to health facilities is essential for users, including pregnant women, it is necessary to identify these barriers and formulate community strategies for the timely referral of these women before or at the start of labor.

Among the limitations of the study, the cross-sectional design does not allow establishing causality given the absence of temporality. Because this study is an analysis of secondary data, there is the possibility of imprecision of the records and memory bias of the respondents. Additionally, there are no data in the literature on some characteristics that have been reported associated with home births, including the distance traveled to health facilities, the presence of complications during pregnancy, satisfaction with health services or the decision of home births by the pregnant woman, which would allow better characterization of the phenomenon studied. Despite these limitations, the ENDES is a nationally representative survey that is widely used in Peru. It uses standardized instruments based on DHS program methodology [44] to study the health status of the Peruvian population and is useful for studying the factors associated with home births in Peruvian women, allowing comparisons with studies from countries with similar methodology.

In conclusion, it was found that less than one-tenth of births occurred at home during the period from 2015 to 2017. The main factors associated with a greater probability of having home births were living in rural areas or the natural Andean and Amazon regions, belonging to the lowest tertile of the wealth index, having a primary educational level, and multiparity. Women in the 25–34 and 35–39 age groups, who had a multiple pregnancy, or had a medium or large-sized newborn were less likely to have home births. Taking into account that home care for childbirth increases maternal and perinatal morbidity and mortality, the development of longitudinal studies focused on some areas with a higher proportion of home births (e.g., rural areas in the Andean and Amazon regions) would provide more information on the determinants of home births and facilitate the development of specific strategies aimed at the needs of the population of childbearing age in order to reduce complications or deaths during childbirth.

Strategies that include training traditional birth attendants for safe birth care and promote home birth care by skilled health professionals could foster the reduction of home delivery care. Likewise, in the communities with the highest proportions of home births, barriers to access to care in health facilities, including transportation and lack of culturally adapted maternal services, should be identified and be addressed to increase the number of childbirth care in facilities due to barriers of access and to improve the availability of emergency care in case of complications arising from home birth care.

## Declarations

### Author contribution statement

Akram Hernández-Vásquez: Conceived and designed the experiments; Performed the experiments; Analyzed and interpreted the data; Contributed reagents, materials, analysis tools or data; Wrote the paper.

Horacio Chacón-Torrico, Rodrigo Vargas-Fernández and Guido Bendezu-Quispe: Analyzed and interpreted the data; Wrote the paper.

### Funding statement

This research did not receive any specific grant from funding agencies in the public, commercial, or not-for-profit sectors.

### Data availability statement

Data associated with this study is freely accessible on the ENDES database is freely accessible and can be obtained at http://iinei.inei.gob.pe/microdatos/.

### Declaration of interests statement

The authors declare no conflict of interest.

### Additional information

No additional information is available for this paper.
